# Endogenous DNA Double-Strand Breaks during DNA Transactions: Emerging Insights and Methods for Genome-Wide Profiling

**DOI:** 10.3390/genes9120632

**Published:** 2018-12-14

**Authors:** Britta A. M. Bouwman, Nicola Crosetto

**Affiliations:** Science for Life Laboratory, Department of Medical Biochemistry and Biophysics, Karolinska Institutet, SE-17165 Stockholm, Sweden

**Keywords:** DNA double-strand breaks, replication, transcription, chromatin looping, topoisomerases, cancer, rearrangements, genome-wide methods, 3D genome structure

## Abstract

DNA double-strand breaks (DSBs) jeopardize genome integrity and can—when repaired unfaithfully—give rise to structural rearrangements associated with cancer. Exogenous agents such as ionizing radiation or chemotherapy can invoke DSBs, but a vast amount of breakage arises during vital endogenous DNA transactions, such as replication and transcription. Additionally, chromatin looping involved in 3D genome organization and gene regulation is increasingly recognized as a possible contributor to DSB events. In this review, we first discuss insights into the mechanisms of endogenous DSB formation, showcasing the trade-off between essential DNA transactions and the intrinsic challenges that these processes impose on genomic integrity. In the second part, we highlight emerging methods for genome-wide profiling of DSBs, and discuss future directions of research that will help advance our understanding of genome-wide DSB formation and repair.

## 1. Introduction

Out of the thousands of DNA insults that hit our cells every day [1], double-strand breaks (DSBs) are among the most deleterious lesions. DSBs can trigger cell death or give rise to structural genomic rearrangements associated with carcinogenesis and other diseases [2,3,4]. The frequency of DSBs is estimated to be 10–50 events per cell per day [5,6]. While exogenous genotoxic insults contribute to this [7,8], in healthy individuals the majority of DSBs is thought to originate from within the nucleus, where DSBs form during fundamental processes such as DNA replication, meiosis, antibody diversification, gene transcription, and—although indirectly—cellular metabolism [1,9,10,11,12,13]. Fortunately, most DSB events are quickly sensed and—orchestrated by the cell’s DNA damage response (DDR) [1,7]—faithfully resolved by two partially redundant repair pathways: homologous recombination (HR) and classical non-homologous end-joining (C-NHEJ) [1,14]. While HR mainly operates in the G2 and S phases of the cell cycle, C-NHEJ is active throughout the cell cycle and is considered the predominant DSB repair pathway in cycling cells. Although C-NHEJ is more error-prone than HR, mistakes of this classical NHEJ repair route are generally minimal [5,15,16]. However, errors become more frequent when alternative end joining pathways (a-EJ) such as microhomology-mediated end joining (MMEJ) engage [17,18,19]. 

In Part I of this review, we describe how DSBs can form during essential DNA transactions—especially during DNA replication, transcription, and 3D genome folding—after which we touch upon the undesired outcomes of DSB repair and their possible consequences. In Part II we attempt to provide a comprehensive overview of the diverse methods that have emerged to profile genome-wide DSB landscapes and we hypothesize how future efforts can help to answer some of the open questions that exist in the field. 

**Part I—Endogenous Causes of DSBs**

## 2. DNA Replication as a Source of Endogenous DSBs

During the S phase of the cell cycle, thousands of replication forks work in a highly coordinated manner to precisely replicate the entire genome exactly once. In the absence of perturbations, a replication fork proceeds undisturbed until it approaches a neighboring fork traveling in the opposite direction. At this point, the two forks fuse, the replication machineries disassemble, and the catenated sister chromatids separate [20,21,22,23]. However, numerous factors can hinder fork progression or interfere with proper execution of the replication program, altogether referred to as replication stress.

### 2.1. Exhaustion of Replication Resources and Disturbed Replication Programs 

Replication forks are forced to slow or stall in a global manner when resources required for faithful completion of replication run out [24]. Essential components such as dNTPs, replication protein A, or chromatin constituents—required for repacking of the replicated DNA and packaging of the newly produced copy—can, for instance, be depleted as a result of perturbed replication timing [24]. Proper licensing of replication origins is crucial for replication precision, and deregulated licensing—which induces systemic replication stress—is believed to contribute to carcinogenesis [25,26]. Activated oncogenes can perturb replication timing by increasing origin firing—which concomitantly depletes replication factors—or by invoking origin re-firing. The latter leads to re-replication, which is particularly detrimental as it not only increases the consumption of resources, but also directly transforms unligated Okazaki fragments into single-ended DSBs [27,28,29,30]. Although oncogene-triggered perturbations of replication timing can lead to genome instability and, as such, play a key role during early cancer development [31,32], oncogene activation can also provoke premature cellular senescence in a process called oncogene-induced senescence, which has been uncovered as an important tumor-suppressing strategy in premalignant cells [33,34]. 

### 2.2. Conflicts with Pre-Existing DNA Damage and Secondary Structures 

**Figure 1:**
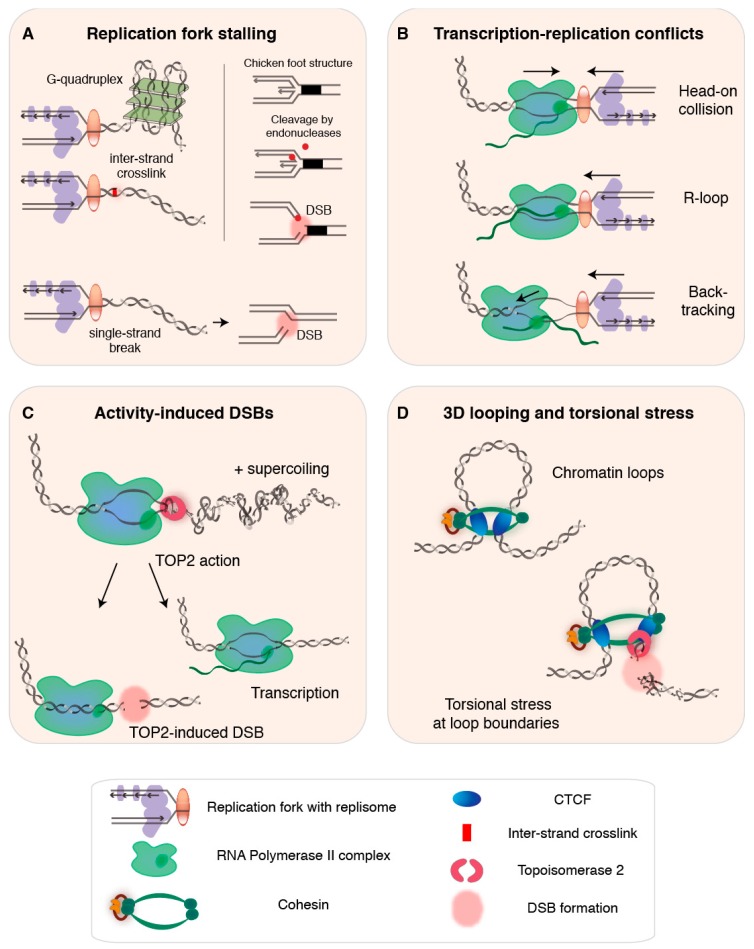
Endogenous DNA double-strand breaks (DSBs) during DNA transactions. (**A**) Left panel: during DNA replication, pre-existing DNA lesions such as G-quadruplexes and inter-strand crosslinks invoke replication stress and cause replication fork stalling. Right panel: an example of how intermediate structures during fork remodeling can lead to formation of a single-ended DSB. Bottom: replication through a single-strand break or nick can result in a single-ended DSB [36,39,41]. (**B**) Transcription complexes obstruct replication fork progression, particularly when transcription-replication encounters are head-on (top) [68], when the nascent RNA has formed an R-loop that stabilizes the RNAPII association with the DNA (middle) [62], and when the RNAPII complex is paused and displays backtracking (bottom, arrow indicates backward sliding of the RNAPII) [59]. (**C**) Transcription-related activity-induced DSBs emerge at sites of topoisomerase 2 (TOP2) action [56,71], which is required to release positive (+) supercoiling building up ahead of the RNAPII complex. TOP2-mediated DSBs enable transcription but can also lead to non-resolved DSBs when repair is escaped or fails [72]. (**D**) Genomic regions involved in 3D genome looping experience torsional stress that requires TOP2 activity to be resolved [73]. As a result, chromatin loop boundaries or anchors may accumulate TOP2-dependent DSBs [74].

Individual replication forks can be brought to stall when an obstacle on the DNA prevents helicase or polymerase activity of the replisome (Figure 1A). If stalling is not properly resolved, the fork can collapse and endanger genome integrity [35,36,37], as we will discuss below in Section 2.4. Pre-existing DNA lesions—including base alterations and strand damage such as inter-strand crosslinks—can cause replication fork stalling [36]. Furthermore, secondary non-B DNA structures that can form at repetitive sequences upon transient strand separation also block fork progression, rendering certain regions of the genome particularly difficult to replicate. Examples of such regions include repetitive telomeric sequences, which can form G-quadruplexes held together by strong Hoogsteen hydrogen bonds [38,39,40,41], centromeres, which are rich in protein-bound heterochromatic AT-rich alpha satellite repeats [42], and tightly compacted, repeat-rich, heterochromatin [24]. In general, obstacles that impede fork progression are believed to have worse consequences when they are encountered on the leading strand, as discontinuous DNA synthesis on the lagging strand may help to bypass obstructions [43,44]. In the next section, we look at another major impediment for replication fork progression: the transcription bubble. 

### 2.3. Transcription-Replication Conflicts, R-Loops, and Backtracking

Transcription bubbles present a natural obstacle for the replication fork [45,46,47] (Figure 1B), and consequences of so-called transcription-replication conflicts (TRCs) are believed to be particularly detrimental when the two machineries encounter each other head-on [46,48,49]. Although DNA replication and transcription are spatiotemporally separated—they globally anti-correlate, occur in distinct cell cycle phases and nuclear sub-compartments, and are presumably subjected to co-orientation bias, which suppresses head-on encounters [21,50,51,52,53,54]—both transactions use the same DNA template, and collisions are inevitable, especially for long genes that require more than one interphase to be transcribed [55]. While TRCs may be rare in healthy human cells, their frequency increases when replication timing is perturbed and in situations in which the transcription machinery lingers [27,56]. 

Genome-wide transcription rates are inherently heterogeneous [57,58], and are influenced by regulatory events such as RNA polymerase II (RNAPII) pausing and/or backtracking [59,60]. Two transcriptional intermediates that have been implicated in TRCs are DNA/RNA hybrids known as R-loops and backtracked RNAPII complexes (Figure 1B). R-loops can form co-transcriptionally when the nascent RNA behind RNAPII anneals back to its DNA template, creating a stable triple-stranded hybrid structure [61,62]. R-loops have been identified in genic regions—at promoters, sites of RNAPII pausing, and transcription terminal regions of genes with short intergenic distances—and, more generally, in regions rich in guanines, expanded trinucleotide repeats, supercoils, and DNA nicks [53,63,64]. Recent work has revealed that R-loops can result from head-on TRCs, whereas co-directional TRCs act as R-loop erasers [65]. Although R-loops can exert regulatory roles [63,66,67], they can also obstruct replication and transcription, and are frequently associated with genomic instability and mutagenic potential, especially when longer stretches of DNA are involved [62,68,69,70]. 

A backtracked transcription complex—composed of a dangling 3’ end of the nascent RNA and an inactive RNAPII that might slide backwards—presents another direct impediment to replication fork progression [59]. Backtracking is associated with promoter-proximal RNAPII pausing—which is considered essential for controlling the accuracy and rate of transcriptional elongation [60,75]—as well as with transcriptional pausing relevant to transcription termination, co-transcriptional RNA folding and processing, and handling of pre-existing obstacles such as DNA adducts and R-loops [59,76]. When not swiftly released or removed, backtracked transcription complexes increase the chance that a replisome switches from the DNA strand onto RNA, which generates a DNA single-strand break (SSB) that can give rise to a DSB during subsequent replication [59]. Although both R-loops and backtracking are frequently related to genomic instability, revealing their direct contribution is complicated by the many co-occurring sources of instability [62]. 

### 2.4. The Cellular Response to Replication Stress and the Impact of Replication Timing 

In general, sensing of replication stress leads to activation of the replication checkpoint, temporary cell cycle arrest, and orchestration of the DDR. Diverse DNA repair pathways can then engage to attempt stabilization of the stalled replication fork—aimed at preventing irreversible fork collapse—and promote removal or repair of blocking impediments or lesions. Ultimately, to restart replication the stabilized fork can be reprimed or, if this fails, replication can be rescued by firing nearby dormant replication origins [24,26,35,37,77,78,79,80,81,82]. Importantly, sustained DDR activation, for example due to failed repair of a lesion, can also lead to apoptosis or senescence [33,83]. 

#### 2.4.1. Fork Stabilization and Restart

Stalled replication forks are predominantly stabilized by components of the HR repair pathway, which also protect nascent DNA at the stalled fork and support repair of DSBs induced by replication stress [24]. Furthermore, the NHEJ pathways C-NHEJ and MMEJ have been identified at stalled replication forks, although they are believed to be mainly implicated in repair of single-ended DSBs at collapsed forks [78,84,85]. Transcription-coupled nucleotide excision repair is thought to assist removal of obstacles that cause replication stress such as R-loops, bulky lesions, and arrested transcription complexes [86,87,88], although rather than stalling, the replisome may also bypass encountered lesions and other impediments with the help of specialized but error-prone DNA polymerases [89,90]. 

After removal of the source of replication stress, restart of the replication fork is believed to occur with the help of HR factors and via repriming mediated by local ssDNA, although the precise underlying fork remodeling and reversal mechanisms remain to be elucidated further (for the main proposed models we refer elsewhere [24,91,92]). While HR is among the most faithful approaches to repair, it can prime error-prone replication restart [78,93]. In Section 5, we elaborate on the consequences of this and discuss more notorious repair processes linked to rearrangements introduced during fork restart. 

#### 2.4.2. DSB Formation from Stalled Replication Forks

When fork stabilization and restart fail the persistently stalled replication fork can collapse, which terminally inactivates the replisome and can result in the formation of a single-ended DSB [24,27,78]. Furthermore, single-ended DSBs can arise at stalled forks that undergo fork reversal or remodeling, for example due to endonuclease-mediated cleavage of chicken foot structures (Figure 1A) or other remodeling intermediates that have been hypothesized to allow DSB-mediated fork restart [77,92,94,95,96]. Single-ended DSBs can also form as the result of replication through nicked DNA [97] (Figure 1A), in case of extensive origin refiring—with or without actual fork collisions (comprehensively reviewed in [30]), or from passively broken ssDNA near stalled forks [24,30,35,98,99]. 

#### 2.4.3. Common Fragile Sites 

DSBs associated with replication stress occur at higher frequency in certain regions of the genome such as common fragile sites (CFSs). CFSs are defined as chromosomal cytobands that appear broken in metaphase spreads following induced replication stress [100,101,102]. CFSs are largely cell type-specific, overlap with recurrent copy number alterations (CNAs) seen in cancer—especially with large deletions—and contain genes with presumed tumor-suppressor activity [102], or belonging to larger functional hubs associated with cancer-related loss of homeostasis [103]. CFS fragility has been attributed to various features that can convey replication stress: repetitive sequence content, premature mitosis, late replication timing or paucity of replication origins, and delayed replication of long active genes [101,102,104,105]. 

#### 2.4.4. Replication Timing and Fragility 

In contrast to CFSs, early replicating fragile sites (ERFSs) represent a different class of fragile genomic regions that replicate early, reside in gene-rich accessible chromatin, and overlap with sites of recurrent translocations and rearrangements observed in cancer [106]. In a large study of somatic CNAs across human cancers, breakpoints of large duplications were found to more often reside in early replicating genomic domains, while deletions and overall CNA levels were enriched in late-replicating chromatin [107]. Similarly, translocation breakpoints in neuroblastoma cells mapped more frequently to early-replicating regions, where the overall breakpoint frequency was estimated to be more than three times higher compared to the rest of the genome [108]. In contrast, point mutation rates are generally higher in late-replicating genomic regions across eukaryotic species [109,110]. 

Although still poorly understood, the connection between replication timing and both fragility and the outcome of erratic repair might in part relate to inherent differences between early and late replication. Regions with early versus late replication timing exhibit roughly mirroring patterns of DNA accessibility, gene activity, and nuclear positioning—in line with A/B compartmentalization. Simultaneously replicating regions may thus reside in closer spatial proximity [111,112]. The more permissive state of chromatin in early-replicating regions likely allows efficient replication, whereas late-replicating—and oftentimes more compact—chromatin requires more origins and thus shorter inter-origin distances [113,114]. The presence of many concurrently active forks in a tighter space in late-replicating regions is thought to present more recombination partners and increase the probability of deletions [110]. In contrast, early-replicating regions are at higher risk of re-replication because a longer period is spent in an already replicated state. As a result, the chances of short-range duplications and rearrangements within the borders of topologically associating domains (TADs)—with intra-TAD rearrangements ranging from 10 to 300 Kb—may be increased [110,115]. Larger and inter-TAD rearrangements (>500 Kb) often seen in cancers [116] are believed to be favored by long-range chromatin contacts between spatially juxtaposed domains with similar characteristics in terms of replication timing and accessibility [107,110,117,118,119].

Although we already briefly touched upon how transcription bubbles contribute to replication stress, accumulating evidence also connects the act of transcription activation itself to elevated levels of DSBs and genomic alterations. In the following section we will therefore focus further on the relationship between transcription and DSB formation. 

## 3. Transcription as a Source of Endogenous DSBs

Besides its share in hampering replication fork progression and generating roadblocks in the shape of R-loops and backtracked transcription complexes, transcription is in itself, in a replication-independent manner, considered to be a source of DSBs [53,56,71,120,121]. As during replication, various types of transcription stress can affect progression of the transcription machinery, and the transient strand separation during transcription is thought to render the non-transcribed strand particularly vulnerable. Although we specifically focus on transcription as a cause of DSBs here, it should be noted that the relationship between transcription and genome instability and mutation is not limited to DSB events, but also encompasses other types of DNA damage. For an extensive overview of transcription-related DNA damage and the connection between transcription and DDR, we refer the reader to an excellent review [121]. 

### 3.1. DSBs Accumulate around Activated Genes 

The genome-wide frequency of DSBs is not only elevated in CFSs, ERFSs, and long, late-replicating genes, but also in accessible chromatin and, in particular, near transcriptionally active genes, with a significant enrichment around the transcription start site (TSS) [122,123,124,125,126]. Transcription-induced DSBs have, for example, been identified near genes responding to signaling invoked by sex hormones and transcription factors in human cancer cells [120], in activated promoters of stimulated neuronal stem/progenitor cells (NSPCs) [127], and DSB levels in mouse brain have been shown to increase upon physiological neuronal activity [128]. In turn, translocation breakpoint clusters, indicative of frequent DSB formation, were found near long, active genes in NSPCs and in activated B cells [124,129]. 

### 3.2. Transcription Activation through DNA Damage

In contrast to previous work that related DSBs and, more generally, DNA damage to the suppression of transcription [130,131], the observed association between fragility and transcription activation suggests that DSBs, in certain situations, may be positively involved in transcription [121,132,133]. Similar concepts have been described before for SSBs generated by type I topoisomerase (TOP1), which emerged at sex hormone-responsive transcribed regulatory elements upon hormone stimulation of prostate cancer cells. In this context, depletion of TOP1 led to reduced transcription of these elements [134]. Furthermore, TOP1 has been implicated in the suppression of long R-loops, by resolving negative supercoiling [62]. 

Examples of transcriptional activation through DSBs—formed as a consequence of DDR pathways—are plentiful [133]. For example, in estrogen-responsive breast cancer cells, DSBs form at estrogen-responding genes through base excision repair (BER), aimed at repairing cytosines deaminated by the action of the APOBEC3B enzyme, which is frequently deregulated in cancer cells [11,121]. In line with the observation that APOBEC3B knockdown led to reduced transcription of the responsive genes, APOBEC3B-induced DSBs are thought to be essential for the recruitment of RNAPII and histone modifications that promote transcription [121]. Furthermore, upon pathogen infection of intestinal cells, endonucleases involved in nucleotide-excision repair (NER) have been identified to cause genome fragmentation. These infection-induced DSBs were shown to be required for activation of the key native immune response, and counteracted infection-associated apoptosis of the infected cells [135,136]. 

### 3.3. Transcription Activation Assisted by TOP2-Induced DSBs 

Transcription-induced DSBs near activated genes in physiological contexts, such as stimulated NSPCs, have been attributed to the action of type II topoisomerase isoforms α (TOP2A) and β (TOP2B), and TOP2-induced DSBs have been suggested to be needed for the transcription of some or all genes [120,127,137,138,139,140] (Figure 1C). This is in line with previous work that found TOP2B-induced DSBs in gene promoters to be required for transcription activation upon hormone stimulation [121,137], and similar observations have since then been described in response to diverse stimuli, including heat shock, serum induction, and various hormonal and neuronal stimuli [11,133]. 

TOP2 forms transient DSBs that help resolve positive DNA supercoiling, which builds up ahead of transcription and replication forks, halting their progression or leading to strand breaks [72,141,142,143,144]. Although the precise mechanism underlying DSB-induced transcription activation remains elusive, TOP2-mediated release of the topological stress brought about by supercoils may be sufficient to stimulate RNAPII processivity [121]. Other postulated models associate both TOP2 and TOP1 activity at transcribed elements to the release of promoter-proximal pausing of RNAPII, allowing transcriptional elongation, or suggest that chromatin alterations upon DSB formation may induce a more transcription-compatible state [121]. 

### 3.4. TOP2 Poisons Are Associated with Therapy-Related Acute Myeloid Leukemias 

Despite the vital function of TOP2 in removing supercoils, its activity is a double-edged sword. On the one hand, TOP2-induced DSBs are thought to be rapidly religated, thereby securing genome integrity [72,145,146]. On the other, the TOP2 religation cycle can occasionally fail, which leaves TOP2 covalently trapped onto cleaved DNA. When the trapped adduct is not removed and repaired via the action of, among others, BRCA1 [147], it can lead to the emergence of a persistent DSB. The danger of this is demonstrated by the TOP2 poison etoposide, a widely used anti-cancer drug that prevents the release of TOP2 cleavage complexes, and thereby generates protein-linked DSBs that block transcription and replication, and eventually kill cancer cells [148]. Despite its successful use in chemotherapy, etoposide has also been associated with the emergence of therapy-related secondary acute myeloid leukemias driven by recurrent genomic translocations between regions coinciding with etoposide-induced DSBs [74,149,150,151]. Of note, etoposide is not the only chemotherapeutic agent that is recognized to increase the chance of secondary malignancies: other TOP2 inhibitors, alkylating agents, and anthracyclines are also associated with an increased risk of developing acute myeloid leukemia, whereas cyclophosphamide treatment increases the risk of bladder cancer [152]. Furthermore, radiotherapy-related secondary cancers can emerge throughout the body depending on the type of irradiation [152,153]. 

In the previous two sections we have discussed relevant work that shows how strongly transcription and formation of endogenous DSBs are interconnected—with and without the interplay with DNA replication. Furthermore, we have seen how transcription activation frequently involves the formation of a transient DSB by TOP2. In the next section we will focus on another functional role of TOP2: resolving topological issues that arise during 3D genome folding. 

## 4. 3D Genome Architecture and DSBs

Topological or torsional stress brought about by DNA overwinding not only affects replication or transcription, in which traversing of the linear genome must be made possible, but it can also impact processes involved in organizing the higher-order three-dimensional folding of the genome. Furthermore, supercoils have been hypothesized to transit through chromatin structure and as such, influence larger stretches of chromatin [144]. In this section, we highlight recent work that has suggested elevated DSB susceptibility at genomic regions involved in 3D genome organization. 

### 4.1. TOP2-Induced DSBs at Chromatin Loop Anchors 

In addition to its binding to gene promoter regions, TOP2B has been identified at cis-regulatory genomic elements bound by CCCTC-binding factor (CTCF) or by both CTCF and cohesin [73,154]. These TOP2B/CTCF/cohesin-bound elements overlap regions uncovered to be relevant for 3D genome organization, such as the borders or anchors of transcriptionally active supercoiling domains [143] and TADS [155,156], as well as the elements involved in regulatory chromatin loops [157]. At these sites, genomic knots and tangles—which require the remodeling activity of TOP2 and perhaps other enzymes—are thought to result from processes proposed to govern chromatin loop dynamics, such as loop extrusion [74,158,159] (Figure 1D). 

Accordingly, chromatin loop borders and loop anchor points are increasingly associated with enhanced levels of DSBs and genomic breakpoints underlying structural variants (SV), and as a result, genome instability and cancer [159,160,161]. While the levels of etoposide-induced DSBs correlate with gene expression levels [126,162], TOP2B-induced DSBs have been reported to be largely transcription-, replication-, and cell type-independent [74], and instead related to the action of TOP2B at the border of chromatin loops [73,74]. However, with chromatin loops forming throughout the genome—often coinciding with active genes [163]—an intertwined relationship between transcription, torsional stress, and TOP2-related DSBs cannot be excluded easily. Indeed, a recent study demonstrated that the frequency of TOP2-induced DSBs enriched at CTCF/cohesin bound loop anchors correlates with expression levels and directionality of coinciding highly transcribed genes—such as those frequently involved in oncogenic translocations in leukemias [164]. Furthermore, inhibition of transcription elongation led to a decrease in DSB levels across transcribed regions and reduced formation of gene fusions, altogether suggesting that both transcription and 3D chromatin folding contribute to TOP2-related genomic instability [164]. 

### 4.2. Intertwined Actions Predispose Regulatory Regions to Fragility 

The topological stress that results from chromatin loop extrusion and other chromatin-related remodeling activities can, besides directly impacting strand integrity, in turn also affect the transcription machinery. As torsional stress can contribute to transcription regulation [142,165], supercoiling and knots emerging during chromatin looping could be involved in, for instance, safeguarding timely termination of transcription, transcription direction, or seclusion of regulatory effects [166]. Furthermore, as mentioned above, the increased susceptibility to indirect breakage at both overwound and underwound DNA [144] can, in principle, affect any region involved in looping, transcription, regulation, or genome condensation. Together with the previous sections, this notion illustrates the complexity that we face when attempting to unravel the mechanisms that underlie the formation of DSBs at a given genomic location, and emphasizes the concept that genomic regions with many concurrent regulatory transactions, such as highly active genes and TAD boundaries in active chromatin, have an inherently higher risk of suffering attacks on strand integrity under physiological as well as diseased conditions. 

### 4.3. Special Cases of Genome Rewiring Require Programmed DSBs

In the sections above, we have covered DNA replication, transcription, and 3D chromatin folding as important endogenous contributors to genome-wide DSB landscapes. However, this overview of endogenous causes is far from complete, as we have deliberately omitted several specialized processes that induce programmed DSBs in specific gene classes or during specific moments in development. We refer elsewhere for excellent literature on programmed physiological DSBs [133], which form in a highly regulated manner during meiotic recombination and the lymphocyte-specific processes V(D)J recombination—which shuffles gene segments to contribute to antigen receptor diversity—and class switch recombination (CSR)—which occurs in mature B lymphocytes and involves a second round of exon substitution to diversify effector antibodies [133,167,168,169]. DSBs essential for V(D)J recombination and CSR are brought about by recombination-activating gene (RAG) endonucleases and activation-induced cytidine deaminase (AID), respectively. Despite the importance of recombination for proper functioning and adaptation of the immune system, the dependency on programmed DSBs poses oncogenic risks [168,170]. For an overview of the protective mechanisms in place to suppress adverse outcomes of DSBs during lymphocyte maturation, we refer to a recent review [171]. 

Although the DDR can in principle repair programmed and accidental DSBs quickly and faithfully, and rescue stalled replication forks to prevent their collapse, DSBs are drivers of most of the structural genomic rearrangements observed in human (cancer) genomes [172]. In the following section, we will summarize some of the main insights into unsuccessful and/or unfaithful DSB repair, and how this can have detrimental effects on genome integrity and stability.

## 5. Adverse Outcomes of DSB Repair 

Imbalances in the regulatory circuitries of the nucleus can cause DNA replication, cell cycle checkpoints, and DDR to go haywire and invoke an avalanche of increasing replication stress, DNA damage, and genomic instability [32,173]. During early carcinogenesis, increasing levels of DSBs—due to enhanced replication stress and transcriptional rewiring—can exhaust the DDR and eventually compromise faithful DSB repair. When C-NHEJ and HR are too slow or simply fail to repair DSBs—for example, due to scarcity or mutational inactivation of one or more of the required factors—alternative mechanisms can engage, of which most are considered to be typically error-prone and hence implicated in the genesis of structural genomic rearrangements [174,175,176]. When DSB repair becomes increasingly erratic during cancer progression, the formation of more oncogenic fusions and CNAs is promoted, which can in turn fuel intra-tumor evolution and heterogeneity. This process is of high clinical relevance, since it can lead to therapy resistance and/or the development of distant metastases [32,173,177]. 

In the following subsections, we first focus on various processes known to be involved in the transformation of a DSB or a stalled replication fork into potentially harmful non-natural junctions. Then, we describe how profiling of the resulting repair signatures—encountered with variable frequencies in cancer genomes—have become a tool to better understand cancer etiology, and predict disease and therapy outcome. 

### 5.1. Mechanisms Underlying Structural Genomic Alterations

Copy number variation (CNV) is widespread in the human genome and underlies natural variation and evolution, but also cancer and developmental and neurological disorders [175,178]. CNVs—large segmental duplications or deletions—require the formation of junctions between sequences that are not normally juxtaposed in the reference genome [175]. The chaotic reassembly of DSB ends or entire genomic fragments—which can give rise to CNVs but also to highly complex genomic rearrangements seen in cancer genomes—has been attributed to complex processes that entail one or multiple rounds of DSB formation and low-fidelity repair, possibly combined with erratic template switching—a strand switch within the same or between distinct replication forks—or replication restart, for example. Below we briefly discuss some of the major processes thought to be involved in generating structural alterations, but for detailed and more complete reviews of proposed mechanisms underlying structural change in the genome we refer the reader elsewhere [174,175]. 

Although HR is considered to be more faithful than C-NHEJ because it utilizes homologous sequences for repair, homology-directed repair is intrinsically mutagenic. In contrast to healthy cells, where HR is strictly controlled [175] and minimized to S-G2 phase and specific recombinogenic processes such as meiotic crossover—which uses allelic HR (AHR)—and V(D)J recombination, HR can jeopardize genomic integrity in oncogenic situations [93,175]. HR repair can cause the formation of genomic rearrangements via abortive intermediates, and at stalled replication forks it can give rise to recurrent CNVs, via non-allelic HR (NAHR), or to non-recurrent CNVs, via error-prone homology-directed break-induced replication (BIR) or single-strand annealing (SSA) [78,93,174,175]. Non-recurrent CNVs can also be formed by NHEJ and other replication-based repair mechanisms, including microhomology-mediated BIR (MMBIR), fork stalling and template switching (FoSTeS), or serial replication slippage (SRS) [174,175]. It has been suggested that multiple rounds of FoSTeS and MMBIR can, for example, underlie the formation of complex rearrangements, while highly complex rearrangements classified as chromothripsis have been hypothesized to emerge during a single catastrophic breakage event, after which the generated chromosomal fragments are erroneously stitched back together, most likely via NHEJ and MMEJ [24,172,174].

In C-NHEJ, DSB ends are repaired without the need for homology, and although the resulting junctions are mostly accurate or have small deletions, free DNA may be inserted or translocations can be formed [175]. In contrast, MMEJ—the best-known a-EJ pathway—joins DSB ends based on microhomology (<25 nt), mediated by the error-prone DNA polymerase θ, which is frequently upregulated in cancer. Its involvement in DSB repair is associated with chromosome rearrangements and small deletions between the microhomologous sequences, giving rise to a typical genomic pattern at the predicted breakpoints [17,18,172]. Rearrangements of larger sections, long-range template switching, and MMBIR are thought to involve the action of other replicative polymerases such as DNA polymerase δ. Other polymerases, including translesion synthesis polymerases—which allow lesion bypass—have also been associated with the induction of local template switching [174]. 

Although most of the mechanisms underlying copy number gains involve replication-based mechanisms, segment amplifications can also arise in a non-replicative manner via breakage-fusion-bridge (BFB) cycles, in which the loss of extensively homologous (sub)telomeric regions induces unstable dicentric chromosomes. It is believed that BFB cycles play a major role in cancer, and the same process has been invoked to explain segmental duplications frequently observed in breast and ovarian cancer [172,175]. 

### 5.2. Repair Signatures in Cancer Genomes 

For many years, patterns of single nucleotide variants (SNVs) have helped uncover distinct mutational signatures in cancer genomes, which has led to improved understanding of the underlying causative processes in certain cancer types. Increasingly, a similar approach is applied to multi-nucleotide structural rearrangements or copy number changes in cancer genomes, based on the identification of junctions between genomic sequences that are not naturally together in the reference genome [179]. Although the computational analyses required to identify complex rearrangement patterns are challenging, successful reconstruction of the genomic junctions in a cancer genome can reveal repair signatures composed of gains, losses, amplifications and rearrangements. As these repair signatures represent scars of impaired repair pathways, studying cancer genomes on a large scale using whole genome sequencing approaches can help to improve our understanding of the molecular history of various cancer types. As certain repair signatures are largely cancer type-specific, they have been harnessed to classify cancers and improve stratification of specific cancer subtypes, as well as to study clonal relationships among metastases and the corresponding primary tumors [172,180,181,182]. 

Although genome-wide profiling of structural rearrangements enables investigation of repair signatures and errors—based on identification of non-linear junctions—these approaches typically reveal past events that have occurred at some point in the history of the cell. In the next section of this review, we change gears back towards the lesions that underlie most of the rearrangements described here, and provide an overview of the various methods that are available for the profiling of genome-wide DSB landscapes.

**Part II—Studying Genome-Wide Fragility Landscapes**

## 6. Methods for Genome-Wide DSB Profiling

With DSBs structurally underlying most types of structural genomic alterations, insight into DNA fragility landscapes and their associated processes—such as transcription and replication, but also recruitment of repair proteins—is essential to broaden our understanding of the genome, particularly in the context of cancer. Over the past few years, various methods for genome-wide DSB detection and identification have been developed, with the aim of obtaining insight into genome fragility and its molecular basis. These methods can be broadly classified into indirect and direct DSB detection methods (Table 1), depending on whether they probe directly for DSBs or for DSB proxies, such as signaling or repair proteins that accumulate at genomic regions hit by DSBs, or products of DSB repair. 

### 6.1. Indirect Identification Based on Association of Recruited or Responsible Proteins

**Figure 2:**
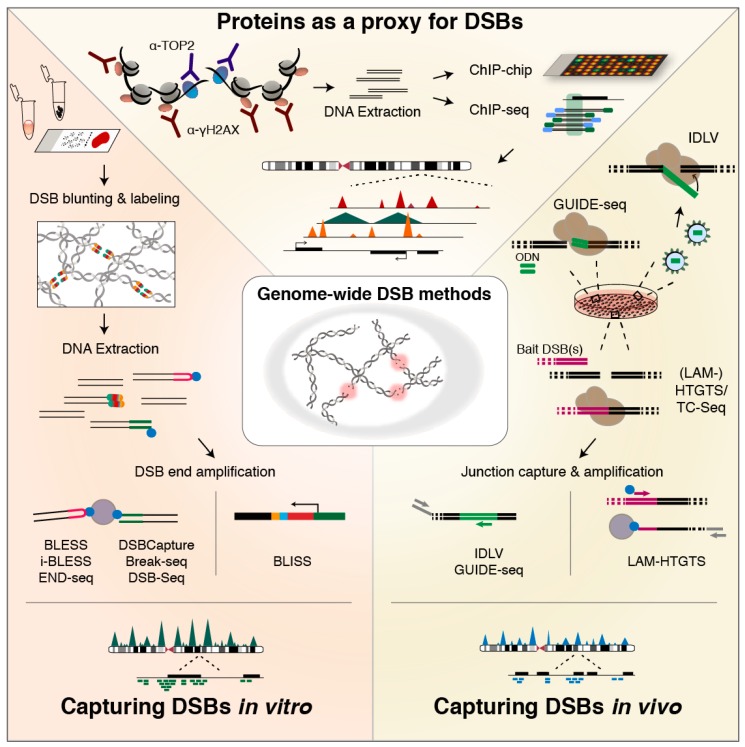
Methods for genome-wide DSB profiling. (**TOP**) Proteins recruited to DSB sites—or associated with DSB formation—serve as a proxy for DSB formation. Chromatin containing the protein of choice is pulled down, and the extracted DNA—representing the underlying genomic regions—can then be analyzed by microarray (ChIP-chip) or high-throughput sequencing (ChIP-seq) [183,187]. The resolution of the generated binding profiles typically depends on the chosen protein. (**RIGHT**) Methods for in vivo capturing of DSBs utilize the non-homologous end-joining (NHEJ) repair machinery of the cell to either incorporate short dsDNA oligos (ODN) (in genome-wide unbiased identification of DSBs enabled by sequencing, GUIDE-seq [194]), or integration-deficient lentiviral vectors (in IDLV capture [195,196]) at the genomic sites of DSBs, or to generate translocation junctions between emerging DSB ends and a bait DSB, exogenously introduced and then induced in the cell (translocation-capture sequencing (TC-Seq) and high-throughput genome-wide translocation mapping (HTGTS) or linear amplification-mediated (LAM)-HTGTS, and derived methods [197,198,199,200]). Afterwards, cells are lysed and DNA is isolated, followed by method-specific approaches for specific amplification or capture of integration or translocation junctions. Subsequently, sequencing libraries are prepared, and sequence reads are aligned to the genome, typically revealing breakpoint clusters genome-wide. (**LEFT**) In vitro methods for genome-wide DSB identification directly label DSB ends with a dedicated adapter—with or without prior DSB end processing—in fixed cells immobilized on a surface (Breaks Labeling In Situ and Sequencing, BLISS [201]) or fixed cell suspensions (Breaks Labeling, Enrichment on Streptavidin, and Sequencing, BLESS [122]), in unfixed cells embedded in agarose plugs or beads (END-seq [202] and i-BLESS [203], respectively), in isolated DNA (DSB-Seq [126]), or isolated DNA in agarose plugs (Break-seq [204]). After labeling, DSB ends are selectively linearly amplified by in vitro transcription enabled by the BLISS adapter in BLISS. In the other methods, DSB ends are captured onto streptavidin beads that selectively capture the biotin-labeled DSB ends, and then amplified. Finally, sequencing libraries are prepared and the resulting mapped sequence reads reveal single DSB ends distributed genome-wide.

The first genome-wide DSB landscape was mapped in yeast, using chromatin immunoprecipitation (ChIP) on a tiled microarray (ChIP-chip) based on antibodies against the phosphorylated form of histone variant H2AX, γH2AX [183]—a ubiquitous component of DSB signaling in eukaryotes [184,185,186] (Figure 2; top). This pioneering work revealed γH2AX enrichment at loci prone to replication fork stalling and breakage, of which half mapped to repressed protein-coding genes [183]. Around the same time, ChIP-chip and ChIP-qPCR [187], as well as ChIP followed by next-generation sequencing (NGS) (ChIP-seq) [188] for γH2AX were applied to human AsiSI-ER cells engineered to conditionally express the AsiSI endonuclease for genome-wide induction of sequence-specific DSBs [187]. These studies revealed that DSB formation triggers large γH2AX domains spreading up to two megabases around the induced DSBs, with active genes protected from γH2AX spreading [187,188]. More recently, ChIP-seq for TOP2B binding was used in addition to γH2AX to indirectly detect DSBs in NSPCs upon exogenous activation. This revealed that TOP2B-dependent DSBs accumulate in the promoter region of early-response genes and are required for their transcription [127]. In recent experiments based on ChIP-exo—in which an exonuclease trims ChIP-ed DNA up to the site of the actual protein-DNA crosslink [189]—TOP2 was found to be positioned at accessible regulatory regions and CTCF/cohesin-bound sites [73]. 

Although highly insightful, exploiting proteins as a proxy for DSBs has the disadvantage of being indirect, and assay outcome may be affected by unspecific binding [190] and spreading of the chosen protein around DSB sites. For example, γH2A.X not only accumulates around DSB sites, but is also recruited to regions with SSBs and to sites undergoing nucleotide excision repair in G1 [186,191,192,193]. To overcome these limitations and enable precise DSB mapping in a genome-wide manner, various approaches to more directly capture and identify DSB ends have been developed, and to comprehensively review these approaches, we distinguish between methods that capture DSBs based on in vivo integration or translocation events mediated by the cell’s DSB repair efforts (Figure 2; right), and methods that directly tag unrepaired DSB ends by in vitro ligation of dedicated adapter sequences (Figure 2; left). 

### 6.2. Methods for In Vivo DSB Capture 

Among the earliest in vivo capture approaches for genome-wide identification of DSBs is integration-defective lentiviral vector (IDLV) capture. IDLV employs in vivo NHEJ-assisted incorporation of lentiviral vectors into DSB sites, which are then amplified by linear amplification-mediated PCR (LAM-PCR) and sequenced [195,196]. IDLV has been applied to detect DSBs introduced by zinc finger nucleases [195], transcription activator-like effector nucleases (TALENs) [196], and CRISPR/Cas9 nucleases [196,205]. Limitations of IDLV capture include integration of the IDLV at varying distances from the actual break site, low detection frequency, sequence bias, low numbers of informative reads, and high costs [194,199]. 

In order to overcome some of these limitations, genome-wide unbiased identification of DSBs enabled by sequencing (GUIDE-seq) was developed [194]. Similar to IDLV capture, GUIDE-seq labels DSBs in vivo via NHEJ-mediated integration of short blunt dsDNA oligodeoxynucleotides (ODNs) at the site of a DSB, after which ODN-labeled genomic regions are amplified and sequenced. GUIDE-seq was established to assess Cas9 and Cpf1 specificity, and is considered to be a highly sensitive and precise method that enables the identification of DSBs that form during a period of several days [194,206]. In GUIDE-seq, identification of a DSB end critically depends on the cell’s NHEJ repair machinery to ligate the blunt ODN to a given DSB end, as well as on the transfection efficiency. 

Rather than identifying DSBs genome-wide via local insertion of an ectopic sequence, several groups have developed techniques that exploit the inherent threat of DSBs being converted into genomic translocations. Translocation-capture sequencing (TC-Seq) [200,207] and high-throughput genome-wide translocation mapping (HTGTS) [197] were developed around the same time, and both methods indirectly identify DSB ends through translocation junctions formed in vivo with a bait DSB, introduced at an ectopically integrated I-SceI endonuclease recognition site. After amplification by conventional PCR or LAM-PCR [198,199], the bait–prey junctions are sequenced. HTGTS and TC-Seq were both applied to identify translocation junctions in B lymphocytes induced for IgH class-switching [197,200], while LAM-HTGTS was also used to assess Cas9 specificity [198]. Moreover, LAM-HTGTS has been harnessed to uncover transcription-associated DSBs in neuronal cells upon mild replicative stress [124,129], and the method has been tailored to uncover antibody repertoires generated during V(D)J recombination in B cells [218]. While the sensitivity of HTGTS methods is biased by the distance of a DSB to the bait—with nearby DSBs translocating with higher efficiencies—LAM-HTGTS was recently further improved by introducing DSB baits on twenty different mouse chromosomes via CRISPR/Cas9. This enabled identification of a more complete set of DSBs as well as appreciation of translocation preferences [208]. 

Although more direct and with higher resolution compared to ChIP-seq, the applicability of IDLV, GUIDE-seq, and HTGTS-based methods is limited by the need for transfection or transduction to enable integration or translocation. This can become especially disadvantageous when aiming to map DSB landscapes in primary cells or tissues, with poor or unknown transfection efficiency and possibly toxic effects of transfection or transduction. Furthermore, as these methods critically depend on active DSB repair, their applicability may be limited when working with cells with impaired DSB repair pathways, such as cancer cells. In IDLV, GUIDE-seq, and HTGTS-based methods, all identified DSB sites have undergone active repair, either locally, with ODN or IDLV sequences being integrated into the broken site, or over larger distances, with translocations onto the DSB bait in HTGTS. This suggests that the identified DSB sites only represent a subgroup of all the DSBs present at a given moment—those that are actively but not faithfully repaired. DSBs that are mostly perfectly and rapidly repaired, for example those induced by TOP2 at promoters, may be largely missed. Furthermore, translocation-based DSB identification methods such as HTGTS can only uncover DSBs in genomic regions that are prone to translocate, and can thus underestimate the actual DSB frequency and the propensity of certain DSBs to give rise to genomic alterations different from translocations. In line with this, the dependency of DSB detection methods on NHEJ repair implies that these methods can miss DSBs repaired through a different pathway [199].

### 6.3. Methods for In Vitro Tagging of DSBs

The first method to directly capture and identify DSBs in fixed cells in situ was Breaks Labeling, Enrichment on Streptavidin, and Sequencing (BLESS) [122]. BLESS captures DSBs by ligating short biotinylated hairpin-like adapters to blunted DSB ends, followed by capture on streptavidin beads, second adapter ligation, PCR and sequencing. BLESS was applied to identify endogenous and replication stress-induced DSBs [122], and to determine the specificity of CRISPR endonucleases [210,212]. Although BLESS does not depend on transfection or NHEJ repair, its labor-intensive protocol requires large amounts of input material and cell fixation, which has been related to the observed high background levels of DSBs [194,199,201]. Recently, two improvements to the original BLESS protocol were described: i-BLESS for highly sensitive unbiased DSB labeling in yeast immobilized in agarose beads [203], and qDSB-seq, enabling normalization of DSB frequency in a sample by spiking in cells in which DSBs were introduced at I-SceI sites [219]. 

Two years prior to BLESS, a method for direct labeling of DNA ends in yeast was published. In contrast to BLESS, damaged DNA immunoprecipitation (dDIP) [214]—among the first methods to generate a nucleotide-resolution map of DNA strand breaks in yeast—does not label DSBs in situ, but in extracted genomic DNA (gDNA). dDIP encompasses end-labeling with biotinylated nucleotides, followed by DNA fragmentation, IP with anti-biotin antibodies, and read-out by qPCR although NGS was also proposed. DNA break immunocapture (DBrIC) applies a similar strategy for immunocapture of DSB ends from gDNA and followed by NGS [215]. Initially, DBrIC was used to demonstrate locus-specific and genome-wide DSBs in human cancer cells. More recently, the approach was harnessed to profile DSB hotspots during the transient genome fragmentation that accompanies post-mitotic chromatin remodeling in mouse spermatids [215,216]. 

Biotin labeling of the 3’ ends of DSBs in isolated high-molecular-weight (HMW) gDNA is also performed in DSB-Seq [126,213], but rather than capture via biotin IP as in dDIP, DSB-Seq exploits the high biotin-streptavidin affinity to capture the labeled ends, which is followed by NGS. Unlike BLESS and most other methods, DSB-Seq can be complemented with the identification of SSBs via SSB-Seq, in which SSBs are tagged by nick translation, captured by immunoprecipitation, and sequenced [126,213]. DSB-Seq and SSB-Seq were applied to map DSBs induced by replication stress using the TOP2 poison etoposide. A major limitation of dDIP, DBrIC, and DSB-Seq is that tagging DSB ends in extracted gDNA may result in increased levels of artificial breaks. This is bypassed in Break-seq, in which isolated chromosomal DNA is embedded into agarose prior to DSB end-repair and biotin labeling [204], after which labeled extracted DNA is captured with streptavidin and sequenced. Break-seq was applied to identify DSB peaks overlapping with DNA replication origins, and to relate DNA fragility to replication fork progression and transcription during and after replication inhibition in yeast [204]. 

Aiming to overcome some of the limitations of BLESS, DSB-seq, and Break-seq, DSBCapture [125] and END-seq [202] were developed. Both methods feature an A-tailing step after blunting of DSB ends, and incorporate the Illumina RA5 adapter sequence directly into the ligated adapter. DSBCapture was applied to identify AsiSI-digested sites as well as endogenous DSBs at regulatory sites and G-rich regions, and revealed that persistent DSBs can be identified under physiological conditions, despite the presence of an intact DDR [125]. In END-seq, besides the adapter improvements mentioned above, DSB labeling is carried out in cells embedded and lysed inside agarose plugs, avoiding cell fixation, which can allegedly cause artificial DSBs [202,220], although a recent study reported very low noise levels in gently fixed samples [203]. END-seq was applied to map AsiSI-induced DSBs and to determine the specificity of DSBs introduced by zinc-finger nucleases, RAG endonucleases involved in V(D)J recombination, and CRISPR-Cas enzymes. Compared to BLESS, END-seq is reported to be more specific and sensitive, and to better preserve the structure of DSB ends [202]. 

To improve the sensitivity and specificity of BLESS, Breaks Labeling In Situ and Sequencing (BLISS) was recently developed [201]. In BLISS, the DSB-labeling adapter contains a T7 promoter sequence, allowing linear amplification of labeled DSB ends by in vitro transcription, and a sample barcode and unique molecular identifier (UMI) to enable high-throughput multiplexing and quantification of DSB ends. T7-mediated amplification helps to overcome the need for streptavidin-mediated pull-down of biotinylated DSB ends, which reduces the required amount of input material. As BLISS can label DSBs in cells or tissue sections immobilized and fixed onto a solid surface, the input requirements are further decreased, making BLISS particularly suitable for studies of genome fragility in clinical specimens [201,221]. BLISS was applied to identify endogenous and exogenous DSBs in cultured cells and tissue sections, as well as to chart the specificity of the RNA-guided endonucleases Cas9 and Cpf1 [201], and Cpf1 variants engineered to recognize alternative protospacer adjacent motifs [222]. Furthermore, BLISS has been used to assess AsiSI-induced DSBs and their repressive effect on gene expression in the DSB inducible via AsiSI (DIvA) human cell line [211]. 

Since the structure of DSB ends is believed to affect DSB repair pathway choice [223], DSB identification approaches that apply DSB end blunting prior to adapter ligation, and especially those that apply formaldehyde fixation [202], are at risk of altering the original and potentially informative structure of DSB ends. To specifically study DSB end structures and to unravel the activities of DSB repair pathways in protecting DSB ends, hairpin capture of DNA end structures (HCoDES) was developed [217], in which DSB ends are treated with ssDNA ligase to form hairpins that allow PCR-based amplification and subsequent sequencing to analyze the precise 5′ end and 3′ end position of both strands. 

### 6.4. On Assay Choice 

From the previous two sections it becomes clear that a variety of methods is now available for generating genome-wide DSB maps, with DSB ends identified at (near-)nucleotide resolution (Table 1). However, navigating the different methods and their differences and advantages can be complicated and overwhelming. Ultimately, assay choice should be steered by the specific research question and the characteristics of the sample to be profiled. To map endogenous DSBs, the differences between mapping DSBs over a period of several hours and based on repair, versus generating a snapshot of all DSBs including very transient ones, as well as intermediates of DSB repair or replication fork remodeling, should be considered. As discussed above, IDLV and especially GUIDE-seq or HTGTS can be great choices for mapping of induced DSB sites, recurrent DSBs and DSB hotspots, but their applicability for the profiling of endogenous genome-wide fragility or fragility related to transcription stress may be somewhat limited by the need for transfection and the time-range required for introduction of the IDLV or ODN sequence or translocation. In relation to this, when repair speed and accuracy at endogenous DSB sites are expected to be high, these methods may specifically miss a considerable amount of endogenous DSB events. Yet, even though genome-wide methods for in vitro tagging of DSB ends are less biased by differences in the type and outcome of the pathways that engage in repair, they can be affected by genome-wide differences in accessibility for DSB blunting and end labeling or adapter ligation, for example. 

When resources permit and when not restricted by cell number or the nature of a clinical specimen, the combination of a genome-wide assay with nucleotide resolution, such as BLISS, with a repair-based assay such as HTGTS, could help to place the results in a broader context. Together, these assays may provide a complementary picture in which both the fragility at a certain moment and then those DSBs able to engage in translocations or insertions over a period of time can be assessed. However, when working with clinical specimens where cell numbers are generally limited and transfectability may be suboptimal and variable between successive samples, it may be favorable to work with cell fixation and in vitro tagging of DSBs.

Another important aspect worth considering is how the generated data will be analyzed to grasp the factors that shape the genome-wide landscape of DSB frequency and distribution in a particular cell type. Fragility scores can for example be correlated to maps of replication timing, transcriptional activity, or chromatin accessibility, preferentially generated in parallel on the same cell type. Furthermore, the frequency of DSB events can be analyzed in light of genome-wide maps of R-loops generated with DNA-RNA immunoprecipitation combined with sequencing (DRIP-seq) [224], binding of transcription or architectural factors created with (exo-)ChIP-seq, and 3D genome folding or nuclear organization, for instance assessed with (capture) Hi-C [225,226] or DamID technology [227]. 

## 7. Concluding Remarks and Outlook

### 7.1. Conclusions and Additional Remarks

In this review, we have highlighted several endogenous processes that can lead to the formation of DSBs—either planned or as a result of local disturbances. Furthermore, although some endogenous DSBs play vital physiological roles—for example, in the context of planned genomic recombination, transcription, or replication fork rescue—any type of DSB represents a threat to the stability of the genome when faithful repair fails. The variety of methods to profile DSBs discussed in Part II, as well as their diverse applications, illustrates the complexity and diversity of the field of DSB identification, especially when one considers that genome-wide nucleotide-resolution methods only represent one of several angles to approach DSB biology. 

#### 7.1.1. Integrative Approaches and Confounding Factors 

In the years to come, to deepen our understanding of variation in DSB susceptibility and cellular implications, integrative approaches will be needed to help decipher how genome-wide landscapes of endogenous DSBs, mapped through one of the methods outlined above (Table 1), are shaped by and/or shape the underlying transcriptome, epigenome, 3D genome, and, possibly, the compartmentalized context of the nucleus. While the genome-wide DSB frequency, distribution [122], and repair rate [228] differ between underlying causative processes, these will in turn also be affected by the spatiotemporally varying architecture of the genome and cell cycle dynamics. Importantly, many factors associated with genome-wide fragility, including DNA replication and transcription, are inherently interconnected, and correlate to underlying genomic and epigenomic features across multiple genomic scales, such as gene density, GC levels, and DNase I hypersensitivity [229]. Hence, understanding the relative impact of a given feature on DSB susceptibility is complicated by potential confounding factors [230], and fragility should be approached as a probabilistic outcome. In the next paragraphs, we will briefly touch upon two features that relate to the observed fragility landscapes and to the possible long-term consequences of repair outcome: clustering of DSBs and repair pathway choice. 

#### 7.1.2. Compartmentalized DSB repair

In addition to certain genomic regions being more prone than others to form DSBs under specific conditions, the possibility of compartmentalized DSB repair and the sequestering of some DSBs to dedicated nuclear regions in mammalian cells is now emerging [231,232,233,234]. For example, using capture Hi-C [225,235] to assess the genomic surroundings and clustering of DSBs induced by the AsiSI endonuclease, clustering was observed in the case of DSBs induced in transcriptionally active genes during G1, and cluster formation was associated with delayed DSB repair [233,236]. Large 53BP1 and γH2AX clusters have also been observed upon mild replication stress in human fibroblasts [237,238], and re-localization of DSBs in heterochromatin to the nuclear periphery—to enable safe HR repair—was recently further elucidated in Drosophila [239,240]. 

DSB repair compartmentalization might allow for spatiotemporal uncoupling of different DSB repair pathways in a cell cycle phase-dependent manner [231,241], potentially acting to suppress genomic rearrangements between juxtaposed repetitive chromosomal domains. Related to this, the choice of DSB repair pathway is controlled by multiple factors, including cell cycle phase and proliferative state, as well as local chromatin composition [209], and by the severity and nature of the DSBs [242]. Interestingly, a recent attempt to quantify the kinetics and fidelity of repair of DSBs induced by the Cas9 nuclease revealed that—in this context—DSB repair is variably slow and surprisingly error-prone [228]. Although these findings may not be directly representative of endogenous DSBs, the presented approach can serve as an inspiration for future studies of repair pathway choice, rate, and fidelity. 

#### 7.1.3. Studying DSB Biology at Ectopically Induced Genome-Wide DSB Sites 

Although endogenous DSBs can be induced by cell stimulation with hormones or transcription factors, or by applying mild replication stress, the effects on fragility are generally not homogenous throughout the cell population, which makes it challenging to draw general conclusions about their effects on transcription or repair. While not valid as endogenous DSBs, the frequently used cell system DIvA [187] and its auxin-inducible degron variant (AID-AsiSI-ER, to rapidly degrade AsiSI and allow DSB repair) [243], have elegantly enabled assessment of the chromatin surrounding the over one thousand AsiSI cut sites, and have helped to pave the way towards better understanding of DSB biology. For instance, a recent study uncovered preferences of different DSB repair pathways as well as chromatin alterations around the most frequently digested AsiSI sites using ChIP-seq [209,243]. While this system presents a powerful tool to study genome-wide effects of DSB formation on transcription of nearby genes, repair, 3D genome organization and many more nuclear processes, a downside is that many of the AsiSI sites in the genome cannot be efficiently cut, and even for the most frequently successfully digested sites, digestion frequency is probabilistic and not uniform across the cell population. 

To our knowledge there is, at present, no method that allows for 100% efficiency of DSB induction at defined sites in the human genome. Although RNA-guided endonucleases are an arguable exception, it has been suggested—as mentioned above—that the cellular response to CRISPR/Cas9-induced DSBs may not be indicative of regular repair of endogenous DSBs [228]. In line with this, also the events observed at the AsiSI sites introduced in DIvA cells may not fully recapitulate the same processes that occur upon endogenous DSB formation. Yet, both of these approaches are highly valuable to broaden our knowledge of DSB biology in the context of the nucleus. 

Furthermore, we envision methods that combine identification of endogenous DSBs with, for example, concurrent assessment of local features such as protein identity or 3D genomic neighborhoods. Another venue of future technology development will be the implementation of combinatorial assays to simultaneously map DSBs and key epigenomic features in the same sample and even single cells, over time. In line with this, single-cell approaches to DSB identification will not only be key to deciphering cell-to-cell variation in fragility landscapes, repair outcomes, and genome folding, but also to helping advance our knowledge of the heterogeneous nature of DSB landscapes in cancer cells and tumor populations underlying tumor evolution. 

### 7.2. Open Questions in the Field and Outlook

Despite the variety of assays available to map genome-wide DSB landscapes, several issues have not been solved so far. As pointed out previously [121], due to the genome-wide nature of these assays, the focus is mostly on sites that form recurrent DSBs. Even when single-cell DSB profiling could be performed robustly, single DSBs would not be easily identified above the background. Furthermore, whether DSB profiling is applied to endogenous or exogenous DSBs, the effect of noise—as well as the absence of a proper notion of what may be considered as background signal—is hard to work around, and low-abundance yet recurrent or pathological DSBs may be masked. Although different assays have their specific caveats, the community would benefit from a reference standard to which all mapped landscapes of DSBs can be compared, and on which novel and existing assays can be tested in addition to regular validation assays [121].

In line with the emerging relevance of repair signatures in cancer genome studies, exploring the levels of ongoing genome fragility by mapping DSBs directly in clinical specimens might provide new insights into the mechanisms that shape cancer genomes and their evolution. Ongoing fragility underlies most of the future CNAs and SVs, and especially in the context of a cancer cell, DSB landscapes may hold clues on highly fragile regions that underlie rearrangements frequently associated with a tendency to metastasize. In line with that, comparing DSB profiles with the landscape of rearrangements at a later timepoint during tumor evolution, as recently attempted [244], will be instrumental for a better understanding of how DSBs are converted into rearrangements that fuel tumor progression. 

## Figures and Tables

**Table 1:** Methods for genome-wide nucleotide-resolution DSB identification.

Method	Detection	Main Features	Sample (Input)	Reported Applications
GUIDE-seq [194]	Indirect	In vivo incorporation of dsODN through NHEJ.	Transfected live cells	Specificity of Cas9 and Cpf1 [194,206]
IDLV capture [195]	Indirect	In vivo random incorporation of integration defective lentiviral vectors, through NHEJ.	Transduced live cells	Cas9 and TALEN specificity [195,196,205]
TC-Seq [200,207], (LAM-) HTGTS [197,199], and3D-proximity based break joining assay [208]	Indirect	Sequencing of translocation products between DSBs ends and a bait DSB, produced via NHEJ.	Live cells treated to induce translocations	Cas9 specificity [198] Replication stress and transcription-associated DSBs [124,129] Translocations in B lymphocytes [197,200] DSB clusters in NSPCs [208]
ChIP-chip and ChIP-seq [183,187,188]	Indirect	Capture of chromatin marked by DSB markers or associated with DSB-inducing enzymes.	Fixed cells (at least 10^7^)	Replication-stress DSBs in yeast [183] AsiSI-induced DSB processing [187,209] Transcription-associated DSBs [210]
BLISS [201]	Direct	In situ blunting and ligation of an adapter containing a T7 promoter, UMI and sample barcode. IVT to selectively, linearly amplify DSB ends.	Fixed cells or tissue sections (at least 10^3^ cells)	Etoposide-induced DSBs, natural DSBs in cells and tissues, and Cas9 and Cpf1 specificity [201] AsiSI-induced DSBs in DIvA cells [211]
BLESS [122] and i-BLESS [203]	Direct	In situ or in agarose blunting and ligation of biotinylated adapters. DSB capture on streptavidin, then PCR amplification.	Fixed cells (at least 10^6^) for BLESS, i-BLESS can use non-fixed cells	Replication stress-induced DSBs in mammalian cells [122] Cas9 specificity [210,212] Rare DSBs in yeast [203]
DSBCapture [125]	Direct	In situ blunting and A-tailing, ligation of adapters with Illumina sequences.	Fixed cells (at least 10^6^)	DSBs at G-quadruplex-rich regions, active genes and transcription start sites [125]
End-Seq [202]	Direct	In vivo blunting and A-tailing in agarose plugs. Labeling with adapters that contain Illumina sequences.	Live cells (at least 10^7^)	AsiSI-induced DSBs, resection mapping, RAG specificity [202] Etoposide-induced DSBs at loop anchors, with and without transcription inhibitors [74]
Break-Seq [204]	Direct	Biotin labeling of DSB ends in HMW gDNA in agarose, then capture and sequencing.	Live cells embedded in agarose (10^6^)	DSB peaks in yeast, to overlap with fork progression during replication stress [204]
DSB-Seq [126]	Direct	Biotin labeling of DSB (and SSB) ends in HMW gDNA, then capture and sequencing.	500 μg HMW gDNA (extracted from 10^8^ cells)	Etoposide-induced DSBs in human colon cancer cells [126] Can be combined with SSB-Seq [126,213]
dDIP [214] and DBrIC [215]	Direct	Biotin labeling of DNA ends in gDNA, then IP and qPCR.	0.5–1 μg extracted DNA	In vitro DSB on a plasmid and induced DSBs and telomeres in yeast [214] I-sceI induced and genome-wide DSBs in HeLa cells [215] DSB hotspots during chromatin remodeling in mouse spermatids [216]
HCoDES [217]	Direct	Hairpin capture of ssDNA-ligated DSB ends, then PCR and sequencing.	10 μg gDNA for ssDNA ligation	5′ and 3′ end analysis of DSBs by RAG, Cas9 and zinc finger endonucleases, and DSBs in G1 repair-impaired lymphocytes [217]
